# The immune system of chicken and its response to H9N2 avian influenza virus

**DOI:** 10.1080/01652176.2023.2228360

**Published:** 2023-07-05

**Authors:** Wenhao Yang, Xiufan Liu, Xiaoquan Wang

**Affiliations:** a Animal Infectious Disease Laboratory, College of Veterinary Medicine, Yangzhou University, Yangzhou, China; b Jiangsu Co-innovation Center for Prevention and Control of Important Animal Infectious Diseases and Zoonosis, Yangzhou University, Yangzhou, China; c Jiangsu Key Laboratory of Zoonosis, Yangzhou, China

**Keywords:** Poultry, chicken, influenza, H9N2, innate immunity, adaptive immunity

## Abstract

Influenza A virus is a negative-sense single-stranded RNA virus that belongs to Orthomyxoviridae family. Based on the antigenic characteristics of hemagglutinin (HA) and neuraminidase (NA) influenza viruses are classified into multiple subtypes. H9N2 belongs to the low pathogenic Avian Influenza Viruses (AIVs) and is one of the widely spread viruses in poultry, which can pose a threat to humans by directly infecting or providing internal genes for various zoonotic avian influenza strains. It has the potential to directly or indirectly participate in becoming an AIV that causes a human pandemic. When the virus enters a host, the innate immune system is activated first by pattern recognition receptors. The cytokines produced at the site of infection recruit innate immune cells and antigen-presenting cells and those cells subsequently transmit antigenic signals to adaptive immune cells (i.e. B cells and T cells), to trigger specific humoral and cellular immune responses. As a result, humoral and cellular immunity can clear virus and infected cells *via* antibody-mediated neutralization and cytotoxicity, respectively. Understanding how chicken immune systems respond to H9N2 is a top priority for effectively controlling the virus’s spread and designing vaccines. In this review, we comprehensively discuss the role of the chicken immune system in defending against H9N2, and clarify the current limitations in understanding chicken immune responses to H9N2 virus, thereby providing potential directions for future research as research on the chicken respiratory mucosal immune system has been stagnant for more than 20 years especially on how the mucosal immune system in chicken responds to avian influenza.

## 1. Introduction

Influenza A virus is a negative-sense single-stranded RNA virus that belongs to Orthomyxoviridae family. Based on the antigenic characteristics of hemagglutinin (HA) and neuraminidase (NA), influenza A viruses comprise 18 HA (1–18) and 11 NA (1–11). While the H17N10 and H18N11 subtypes are found exclusively in bats, the natural hosts of all other HA and NA subtype viruses are wild waterfowl and sea birds (Wu et al., 2014). Avian influenza viruses (AIVs) can be roughly divided into highly pathogenic AIVs (HPAIVs) and low pathogenic AIVs (LPAIVs) based on their pathogenicity in chickens and molecular markers of the HA protein. The HA protein of HPAIVs contains multiple cleavage sites that can be cleaved by an endogenous cellular furin-like protease, leading to severe systemic infection and high mortality in chickens. By comparison, the HA protein of LPAIVs contains mono-, di-, or occasionally tri-basic cleavage sites, which can only be cleaved by extracellular trypsin-like proteases.

H9N2 belongs to the LPAIV and was first identified in turkeys in the U.S.A. state of Wisconsin in 1966 (Homme and Easterday, 1970). In the decades since, H9N2 has spread widely in different parts of the world, including Africa, Asia, Middle East, and Europe (Alexander, 2007). Originally, the virus did not initially infect chickens, only turkeys and occasionally quails (Wu et al., 2009). Due to its segmented genome and error-prone RNA polymerase, AIVs are prone to gene mutation and gene reassortment, processes that often allow them to overcome limitations in the range of potential hosts (Webster et al., 1992). Gene reassortment poses a greater impact for avian influenza. Amid the continuous evolution of H9N2, its host range has also expanded from mostly turkeys to also include chickens (Fioretti et al., 1999), pig (Cong et al., 2007), ferret (Ku et al., 2014), dog (Sun et al., 2013) and even man (Guo et al., 1999). H9N2 actively participates in gene reassortment and provides complete internal genes for other novel influenza viruses, including H5N1 (Guan et al., 1999), N7H9 (Lam et al., 2013), H10N8 (Chen et al., 2014) andH5N6 (Shen et al., 2016). Most of those viruses are zoonotic and make humans highly susceptible to related diseases and death. At present, the primary AIV subtypes in commercial chickens and ducks have transformed from H5N6 and H7N9 to H9N2, and nearly all H9 AIV strains prefer human-type receptors (Bi et al., 2020). Compared with viruses of wild bird lineage, the H9N2 viruses of poultry lineage have been shown to preferentially recognize α-2, 6-linked sialic acids, and replicate in higher titers in mammalian cells and mice (Guo et al., 2021). Study has shown that H9N2 can infect civet cats and Asian badgers - causing respiratory symptoms in the latter - and may be at play in cases of human-to-wildlife virus transmission (He et al., 2022). At present, the prevalence of the H9N2 virus in humans is also not promising. Although there are fewer reported cases of human infection and illness or death caused by H9N2, from 2014 to 2016 the H9N2 AIV’s seroprevalence rate among healthy poultry workers reached 11.2% in many provinces of China, and with a rising trend (Li et al., 2017; Quan et al., 2019). Other countries have also reported high seroprevalence rates of H9N2 among poultry workers, including Nigerian (Okoye et al., 2013), Romanian (Coman et al., 2013), Indian (Pawar et al., 2012), Cambodian (Blair et al., 2013), Vietnamese (Uyeki et al., 2012), Iranian (Alizadeh et al., 2009), and Pakistanian (Ahad et al., 2013). H9N2 is considered as the AIV with the greatest potential to cause a cross-species pandemic (RahimiRad et al., 2016).

Chickens, as the largest host population for H9N2 AIV, play a critical role in the transmission and evolution of the H9N2 subtype. As vertebrates, avian species have many similarities with mammals in their immune systems and immune responses to invading pathogens. The avian immune system also exhibits an impressive diversity, including the bursa of Fabricius, a unique B cell-generating organ, and the Harderian gland, a local immune secondary lymphoid organ (Bang and Bang, 1968; Swayne and Kapczynski, 2008), as well as a lack of lymph nodes and the pattern recognition receptor RIG-1. Despite numerous differences in the immune systems of chickens compared with mammals, chickens’ immune genes are generally consistent with those of mammals based on genome sequencing. Like mammals, chickens’ innate immunity also drives adaptive immunity, which is essential for resisting pathogens and clearing pathogens and providing immune memory. Against that background, this review article primarily focuses on summarizing the immune response mechanism of chickens to H9N2 AIV infection, including their innate immunity and adaptive immunity, and provides information for exploring H9N2’s pathogenesis and immune protection mechanisms.

## 2. Innate immune response to H9N2 AIV infection in chicken

The innate immune system is considered to be the first line of defense, which can combat pathogens as soon as they invade the body. The innate immune system includes (1) physical and chemical barriers; (2) innate immune cells, including dendritic cells, natural killer (NK) cells, and macrophages; (3) pattern recognition receptors (PRRs) expressed by innate immune cells; and (4) complementary proteins and cytokines. Compared with adaptive immunity, innate immunity is immediately involved in resisting pathogens and the first to appear in the body.

### 2.1. Pattern recognition receptors (PRRs)

**Figure 1.**
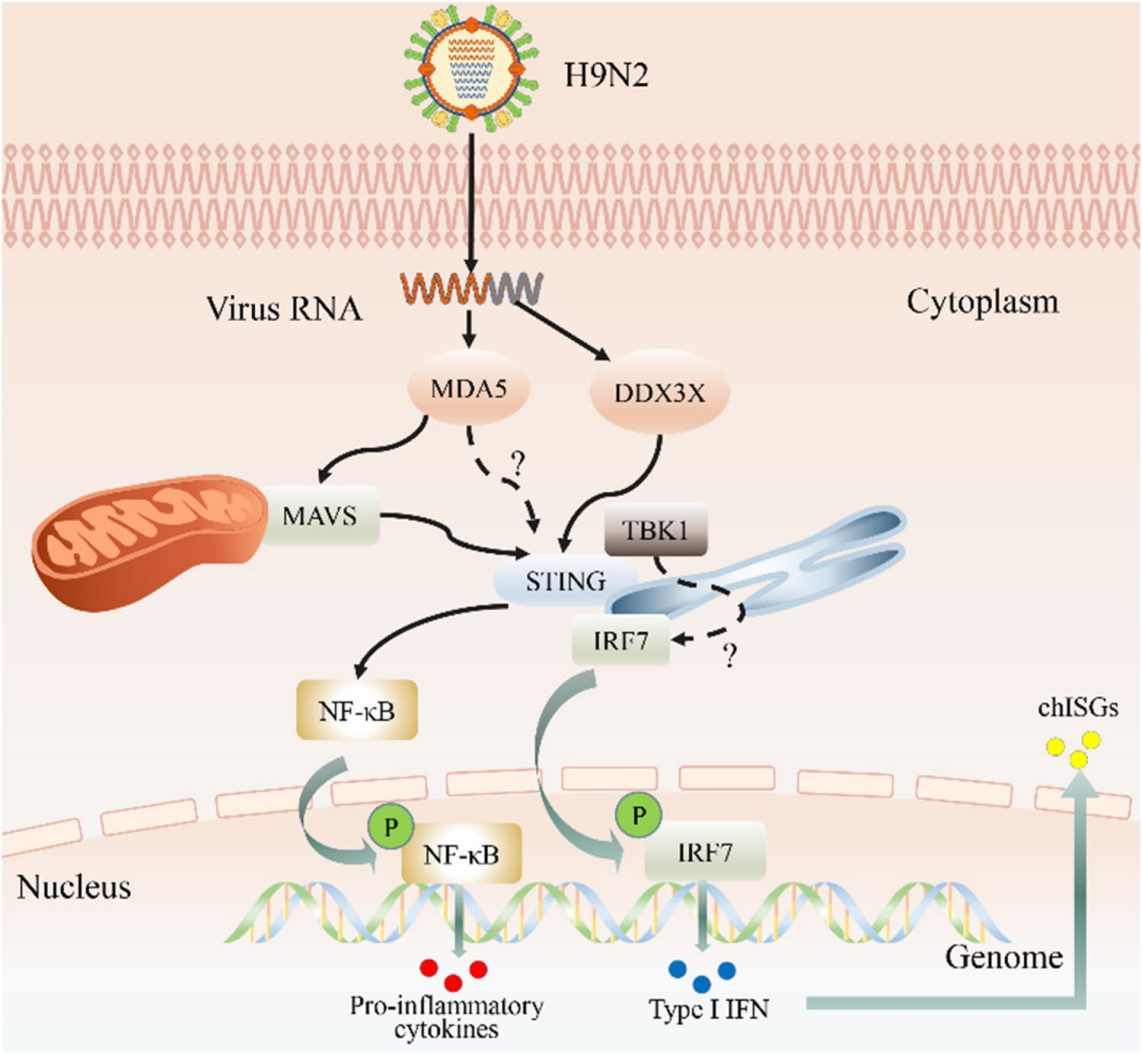
Innate immune response against H9N2 AIV infection in chicken. H9N2 virus is recognized by melanoma differentiation-associated gene 5 (MDA5) and asp–-glu–-ala–-asp (DEAD)-box polypeptide 3 X-linked (DDX3X) which trigger downstream pathways through the stimulator of IFN genes (i.e. STING) and the mitochondrial antiviral-signaling protein (MAVS), respectively. Although MAVS also activates STING after receiving the upstream signal, what remains uncertain is whether MDA5 directly activates STING. In any case, STING activates the transcription factors IFN regulatory factor 7 (IRF7) and nuclear factor kappa B (NF-κB) by orchestrating the assembly of TANK-binding kinase 1 (TBK1). even so, the interaction between TBK1 and IRF7 also remains unclear. Once activated, IRF7 and NF-κB translocate into the nucleus and are phosphorylated, which further stimulates the transcription of type 1 interferons and proinflammatory factors. In turn, type I IFNs stimulate the production of the IFN-stimulated gene factor (ISG) by both autocrine and paracrine signaling through cognate type I IFN receptor recognition.

PRRs are the sentinels of innate immunity, primarily expressed on the surface or in the cytoplasm of monocytes, macrophages, dendritic cells (DCs), and non-immune cells (e.g. fibroblast cells, endothelial cells, and mucosal epithelial cells), that recognize the pathogen-associated molecular patterns (PAMPs) and damage-associated molecular patterns of pathogens (DAMPs) (Takeuchi and Akira, 2009). There are four major families of PRRs in chickens: Toll-like receptors (TLRs) (Nang et al., 2011), retinoic acid-inducible gene I (RIG-I)-like receptors (RLRs) (Liniger et al., 2012), nucleotide-binding oligomerization domain (NOD)-like receptors (NLRs) (Chothe et al., 2020; He et al., 2021), and, most recently discovered, cyclic-GMP–AMP synthase (cGAS) (Li et al., 2020). Among differences in innate immune signal pathways between chickens and mammals, chicken TLR21 is a functional homologous surrogate for mammalian TLR9 and may exhibit a relatively broad recognition of species (Brownlie and Allan, 2011; Dalpke et al., 2006). Beyond that, the important receptor molecule, RIG-I, in the RLR pathway is absent in chickens (Zou et al., 2009). Instead, RNA ligands are recognized by other cellular sensors such as melanoma differentiation-associated protein 5 (MDA5) and TLRs. Liniger et al. were the first to demonstrate that chMDA5 is involved in chIFNβ’s induction against infection with H5N1 AIV (Liniger et al., 2012), as later confirmed for H9N2 (Hayashi et al., 2014). In addition, like RIG-I in mammals, the NS1 protein of AIV has been shown to inhibit chMDA5 signaling (Liniger et al., 2012). By knocking out MAD5 and TLR3, MDA5 has been identified as the primary sensor for sensing RNA ligands in chicken DF1 cells, with TLR3 being the secondary sensor (Lee et al., 2020). Moreover, MDA5 can complement the lack of RIG-I by interacting with the stimulator of IFN genes (STING) to form the MDA5-STING-IFN-β pathway, which confers a strong antiviral state against RNA virus (i.e. H9N2), while STING activates both NF-κB and IFN regulatory factor 7 (IRF-7) transcription pathways to induce type I IFN and IFN-stimulated genes (ISGs) (Figure 1) (Cheng et al., 2015). The location-specific upregulation of TLR3, TLR7, TLR21, and MDA5 in oviducts has been observed in oviducts of laying hens infected with H9N2 (Wang et al., 2015). Furthermore, the expression of TLR3 and NLRC5 gene was found to be upregulated in DCs derived from chicken bone marrow infected with H9N2 virus in RT-Quantitative Real-time PCR (qPCR) analysis (Liu et al., 2020). NLRC5 can be activated by various LPAIVs and HPAIVs in primary chicken lung cells and chicken macrophage cell lines (HD11). However, the siRNA-mediated knockdown of NLRC5 has been shown to inhibit the replication of influenza virus, which suggests that NLRC5 acts as a negative feedback regulator during AIV infection in chickens (Chothe et al., 2020). In addition, the Asp–Glu–Ala–Asp (DEAD)-box polypeptide 3 X-linked (DDX3X) may also participate in the RNA virus-mediated IFN-β signaling pathway under the stimulation of H9N2 (Niu et al., 2019).

### 2.2. Dendritic cells (DC)

DCs play a crucial role in initiating adaptive immunity and act as the bridge between innate immunity and adaptive immunity. DCs can process antigens and present antigenic to T cells in the form of the antigenic peptide-majority histocompatibility complex (MHC) molecular complex and the co-stimulatory signal. They also have the unique ability to activate naive T cells. Immature DCs have a powerful phagocytic capacity. When stimulated by inflammatory responses and pathogens, they migrate along the vessel wall toward T cells while expressing high levels of chemokine receptors such as CCR7 (Liu et al., 2021; Wu et al., 2011). In turn, DCs stimulate the activation and proliferation of T cells by secreting proinflammatory cytokines such as IL-12, IL-18, and TGF-β (Gutcher and Becher, 2007), and by cooperating with the first and second signals of antigen presentation. During recognition phagocytosis of pathogens, and antigen presentation, immature DCs gradually transform into mature DCs with reduced phagocytic capacity and the increased expression of MHC molecules, costimulatory molecules, and the chemokine receptor CCR7. DCs play an irreplaceable role in transmitting immune signals to T cells. Compared with DCs in mice and humans, chicken DCs have seldom been investigated. It was not until 2009 that Wu et al. cultured chicken DCs *in vitro* for the first time and initially proved their function, which can mature into a T helper type 1 (Th1)-promoting phenotype *via* the stimulation of LPS or CD40L (Wu et al., 2010).

Few studies have investigated the role of DCs in chicken immune systems during H9N2 AIV infection. They mainly focused on proteomics and transcriptomics or how to improve H9N2 AI vaccine efficacy by targeting the stimulation of DCs. Even so, a gene ontology (GO) enrichment analysis of global gene expression revealed that 130 and 120 GO terms were up- and downregulated, respectively in chicken DC cells after H9N2 AIV infection. These terms were primarily enriched in cellular components, molecular functions, and biological processes (Liu et al., 2020). Other authors have also reported transcriptomic and microRNA data in chicken DCs infected with the H9N2 virus (Liu et al., 2020; Yang et al., 2020). When DC stimulators such as CpG DNA, DC-binding peptide (DCpep), and antibody to the CD83 receptor were used as potential adjuvant for H9N2 AI vaccine, CpG DNA was found to effectively activate TLR21 in chicken bone marrow-derived DCs *in vitro*, increase the expression of IRF7 and tumor necrosis factor (TNF) receptor-associated factor 3 (TRAF3), and further stimulate the expression of IL-1, IL-6, and IL-10 (Lin et al., 2014). In another work, virus-like particles expressing the haemagglutinin–neuraminidase of Newcastle disease virus and the HA of H9N2 were decorated with DCpep. They activated chicken DCs *in vivo* and promoted sIgA secretion and splenic T cell differentiation, and better inhibited the viral shedding of H9N2 when administered intranasally (Xu et al., 2020).

CD 83 is a marker of early DC activation and plays an important role in B cell antibody production against influenza A viruses (Akauliya et al., 2020; Lee et al., 2012). When HA antigen was fused to single-chain fragment variable (scFv) antibodies specific to the CD83 receptor, it improved the expression level of IFN-γ, IL6, IL1β, IL4, and CxCLi2 mRNA in chicken splenocytes *in vitro*, as well as inhibited viral shedding after H9N2 challenge in chickens (Shrestha et al., 2021). Another study showed that scFv antibodies specific to chicken CD11c receptors, when fused to H9 HA protein to form trimers, can improve the immunogenicity of HA (Shrestha et al., 2021). Moreover, H9N2 has been able to replicate in the monocyte-derived dendritic cells (MoDCs) of chickens and produce progeny viruses. In that study, compared with H5N1, relatively low levels of IFN-α, IFN-β, and IFN-γ mRNA expression were induced after H9N2 AIV infection of MoDCs. The same trend was observed for TLRs 3, 5, 15, and 21 and death rate of MoDCs. However, H5N1 caused more severe damage. Those findings suggest that MoDCs play an important role in immune deregulation in chickens during H5N1 and H9N2 AIV infection (Kalaiyarasu et al., 2016).

### 2.3. Macrophage/monocyte cells

As important members of the innate immune response, macrophages are generally considered to be the first cells to encounter pathogens (Schat et al., 2014). They can perform various functions such as clearing pathogens, modulating innate immune responses, activating adaptive immunity, and maintaining tissue homeostasis (Taylor et al., 2005). Previously, it was thought that macrophages differentiated from monocytes in blood vessels after infiltrating tissue through the vascular endothelium. However, a more recent study has shown that macrophages in tissues are not terminally differentiated; on the contrary, the differentiation phenotype of macrophages depends on the type of tissue and is influenced by the tissue environment that regulates their gene expression (Taylor et al., 2005). In mammals, macrophages are susceptible to infection with certain influenza viruses and express viral proteins, but cannot produce progeny viruses. This phenomenon has also been observed in chickens (Mock et al., 1987; Van Campen et al., 1989). Nevertheless, some studies suggest that certain influenza viruses can replicate productively in the macrophages of different mammals (Cline et al., 2017). These studies indicate that the ability of influenza viruses to replicate productively in macrophages is strain-specific.

Macrophages are generally divided into two major phenotypes—M1 and M2—polarized by IL-4 and/or IL-13 and IFN-γ, respectively. The macrophages activated by IL-4 and/or IL-13 are involved in regulating the Th2 immune type, which is responsible for controlling parasitic infection, promoting tissue remodeling, and modulating immunity (Murray et al., 2014). The macrophages activated by IFN-γ are involved in regulating the Th1 immune type, which is characterized by the high production of reactive nitric oxide (NO) and oxygen intermediates, high expression levels of proinflammatory factors, and strong microbicidal and tumoricidal activity (Sica and Mantovani, 2012). In 1989, Jeurissen et al. reported that macrophages appear in chicken embryos on embryonic incubation day (EID) 12 in the liver and EID 16 in the spleen (Jeurissen and Janse, 1989). The secretion of NO and reactive oxygen is considered to be an important mechanism for the clearing of pathogens by activated macrophages in mammals (Babior, 1992; Babior, 2004), which has also been demonstrated in chickens (Palmquist et al., 2006; Qureshi, 2003). Interestingly, IL-4 can stimulate NO production in chicken HD-11 cells, which has not been observed in mammals (Paoliello-Paschoalato et al., 2005; Xu et al., 1999), and this effect is stronger than IFN-induced NO production. However, when macrophages were stimulated by microbial agonists form bacteria and viruses, IL-4 showed a strong inhibition of NO secretion (He et al., 2011). The mechanism of IL-4’s bidirectional regulation of NO production by macrophages could elucidate the function of chicken innate immunity in response to disease invasion, which occurs at stage of innate immunity regulation by macrophages. Research has shown that IL-4 expression is downregulated after HTC macrophage cell line infection with H9N2, which plays a key role in regulating Th2-type immune responses (Xing et al., 2008). However, other studies have reported contrasting results, with IFN-γ and IL-4 significantly upregulated in DH11 cells infected with H9N2 at 12 h and 24 h, respectively (Chu et al., 2020). In mammals, alveolar macrophages exhibited polarization toward the M1 phenotype 4 h after H9N2 AIV infection, with the upregulation of M1-associated marker genes STAT1, TNF-α, MCP1, INOS, IL-6, and IL-12. After 8 h, however, the macrophages tended to polarize toward the M2 phenotype as indicated by the upregulation of M2-associated marker genes IL-10. After 24 h, they showed an immunosuppressive phenotype characterized by the downregulation of both M1- and M2-associated genes STAT1, MCP1, IL-12, and MGL1 (Zhao et al., 2014). The expression levels of cytokines in macrophages after H9N2 AIV infection are unclear and divergent and the mechanism of how macrophages regulate T cells through cytokines in chickens remains unknown.

Influenza viruses can activate anti-apoptotic P13k–Akt signals at the early and middle (5–9 h) post infection (p.i) stages of infection and thereby preventing cells from apoptosis and ensure viral replication to produce progeny viruses in mammalian cells. However, at late stages of infection, both p53-dependent and alternative p53-independent apoptotic and/or necrotic signals were activated to release the progeny virus from the cells (Zhirnov and Klenk, 2007). Compared with H6N2, H9N2 induced severe apoptosis at 18 h after infecting macrophages (Xing et al., 2008). The authors also demonstrated that the NS1 protein of H9N2 could inhibit Fas/Fasl-mediated apoptosis at 10 h and 24 h after infecting HTC macrophage cell line, thereby improving the infectivity of chicken macrophages (Xing et al., 2009).

### 2.4. Natural killer (NK) cells

NK cells are considered to play an important immunomodulatory role as effector cells in innate immunity. NK cells can kill target cells by MHC-independent and antibody-dependent cytotoxicity (ADCC), and produce various cytokines, including interferon γ, TNF-α, and granulocyte–macrophage colony-stimulating factor (Straub et al., 2013). Perforin and granzymes are effector granules that are mobilized and secreted by activated NK cells to exert cytotoxic effects and induce the apoptosis of target cells (Kägi et al., 1994; Voskoboinik et al., 2006). In mammals and chickens, accumulating evidence suggests that NK cells play an important role in pulmonary immune responses against respiratory disease (Jansen et al., 2013; Rogers et al., 2008; Vervelde et al., 2013). In chicken embryos, NK cells first appear at 13–16 d in the spleen, where they express CD8 molecules and lack TCR (Alkie et al., 2019; Göbel et al., 1994). In adult chicken, classical-like NK cells mainly exist in the intestine, whereas other organs or tissues mainly have NK T cells. Unlike mammalian NK cells which account for about 20% of blood lymphocytes, NK cells in chickens appear in very low proportions in the blood, spleen, and cecal tonsils, namely from only 0.5 to 1.0% (Rogers et al., 2008). CD3^−^CD56^+^CD16^+^ and CD3^−^NKp46^+^ are often used to define NK cells in man and mice, respectively (Cong and Wei, 2019). But there are currently no commercial mature antibodies available to identify NK cells in chicken. As an alternative, some researchers have developed and used antibody 28-4 with CD3 to identify NK cells (Abdolmaleki et al., 2018; Meijerink et al., 2021).

NK cells are involved in the early immune response against viral infection. In humans, the NKp46 and NKp44 receptors of NK cells can directly recognize the HA protein of influenza viruses, meaning that the NK cells themselves can directly lyse cells infected with such viruses^-^ (Arnon et al., 2001; Ho et al., 2008; Mandelboim et al., 2001). In mouse models, NK cells play a dual role in responding to influenza virus infection, with deleterious effects on the host at high doses and the opposite at low doses (Stein-Streilein and Guffee, 1986; Zhou et al., 2013). In chicken, research has revealed that the ratio of CD3^−^CD8α^+^ cells in both the lung and peripheral blood mononuclear cell (PBMC) was upregulated 3 d after H9N2 AIV infection, as were lung CD107^+^ cells, which suggests that lung NK cells were indeed activated. NK cells thus play an active immune role in defending against H9N2 AIV infection (Jansen et al., 2013).

## 3 Adaptive immune response to H9N2 in chicken

Although innate immune response can resist the invasion of influenza viruses, adaptive immunity is ultimately required for virus clearance, a complex process that involves multiple immune cell systems, including DCs, B cells, T cells, macrophages, and NK cells (Figure 1), as well as cytokines.

### 3.1 T Cells

#### 3.1.1. CD4 and CD8 T cells

**Figure 2.**
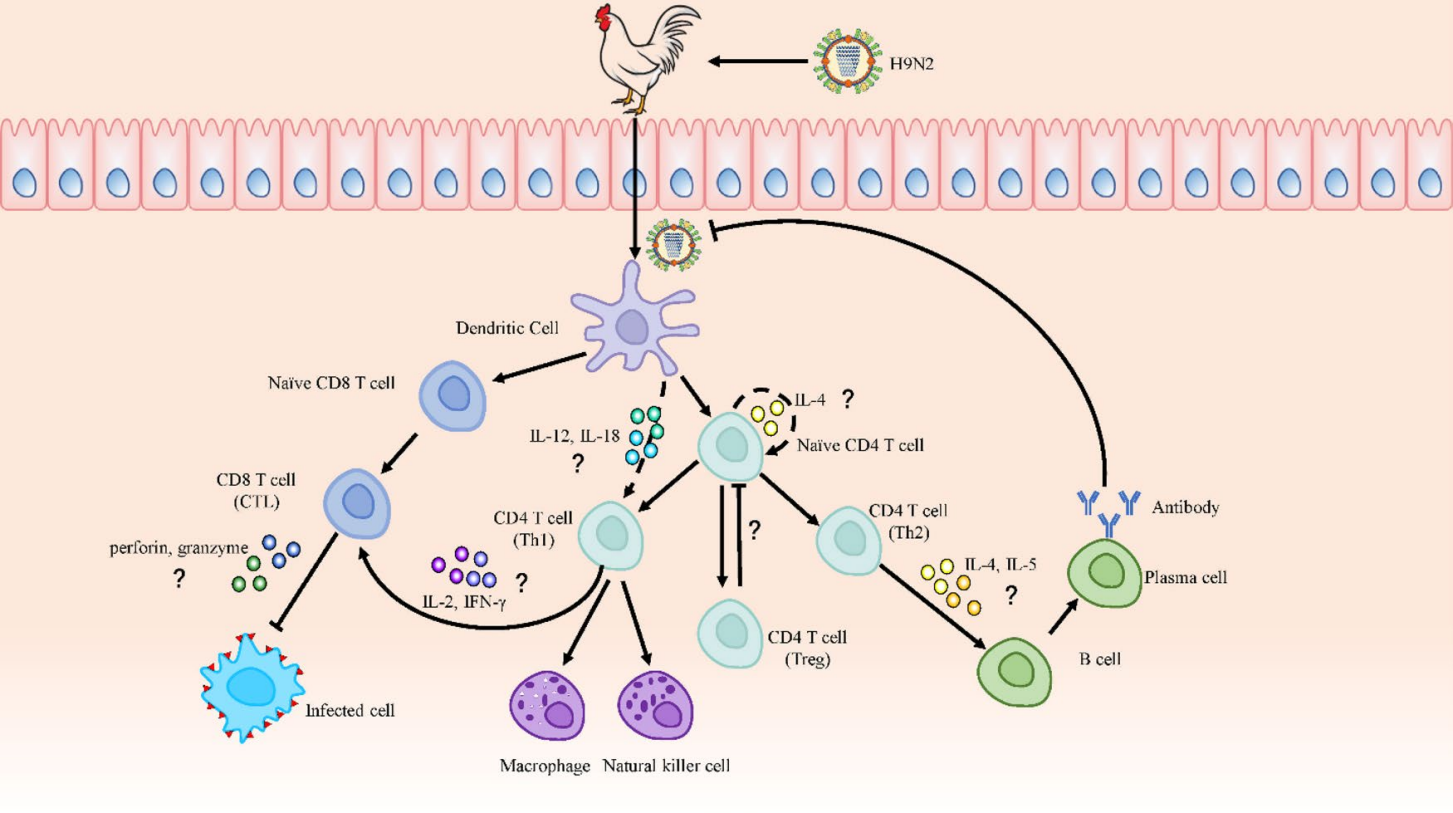
The Possible adaptive immune response against H9N2 AIV infection in chicken. The mechanisms of cytokines and signaling molecules in adaptive immunity remain largely elusive in chickens, so relevant studies of mice and humans are cited to furnish explanations. After the H9N2 virus infects chickens, it is recognized by antigen-presenting cells (APCs), among which DCs are the most important. DCs present extracellular and intracellular antigens to naive CD4 and CD8 T cells after capturing and processing antigens, respectively. CD4 T cells subsequently differentiate into Th1, Th2, and Th17 cells. Th2 cells are induced by autocrine IL-4 from naive CD4 T cells. Th1 cells are induced by IL-12 and IL-18 secreted by DCs. Th1 cells promote the activation of cytotoxic T lymphocyte (CTL) cells, which induce cytotoxicity by secreting IL-2 and IFN-γ, and, then granzyme and perforin are important effector molecules of CTL cells. Meanwhile, Th2 cells secrete IL-4 and IL-5, which promote the production of virus-specific antibodies by B cells.

T cells, which mature in the thymus, mediate adaptive cellular immune responses and play an important auxiliary role in thymus-dependent antigen-induced humoral immunity. Thus, T cells play a central role in adaptive immunity. T cells require three signals to work together to make a productive response and to avoid death and/or immune tolerance (Curtsinger and Mescher, 2010). The first signal is the recognition of the antigen by naïve T cells through the specific binding between the surface TCR and the MHC molecule of APCs. The second signal is the interaction of multiple pairs of costimulatory molecules, mainly CD28, on the surface of T cells and APCs. The third signal is inflammatory cytokines secreted by APCs and critically determining the direction of T cell differentiation. The generation of Th1 cells is mediated by the synergy of IL-12 and IL-18 (Figure 2), whereas TGF-β secretion can polarize naive cells toward a regulatory phenotype with Regulatory T cells or an auto aggressive phenotype with Th17. In the latter case, the production and maintenance of Th17 cells also requires the cytokines IL-6 and IL-23. T cells secrete IL-4 in an autocrine manner and act on themselves to polarize Th2 cells, a process induced by the interaction between APCs and T cells (Gutcher and Becher, 2007). Th1 cells mainly respond to the infection of intracellular pathogens, including intracellular parasitic bacteria and viruses, by secreting IFN-γ, IL-2, and TNF-α. Meanwhile, Th2 cells mainly respond to the infection of extracellular bacteria, fungi, and parasites, by secreting IL-4, IL-6, and IL-10 (Szabo et al., 2003).

In chicken, T cells first appear in the thymus of embryonic chicks at 10–12 d of development (Seto, 1981). Study has shown that although the T cells of 1-d-old chicks are phenotypically mature, they have no immune function and cannot achieve the immunity of adult chicken until they are one week old (Lowenthal et al., 1994). In chicken, IL-12 and IL-18 produced by APCs also are important cytokines in the differentiation of CD4 T cells to the Th1 type (Degen et al., 2004; Guo et al., 2013; Schneider et al., 2000) and regulate IFN-γ production (Szabo et al., 2003). Although humoral immunity mediated by antibodies can prevent AIVs infection, they are strain-specific. T cells, especially CD8 T cells, can provide a broad range of cellular immunity to different subtypes of AIVs (McMichael et al., 1983; Sridhar et al., 2013). In previous research, T lymphocytes from chickens infected with H9N2 virus protected naive chickens from a lethal H5N1 virus challenge (Seo and Webster, 2001). In addition, lymphocytes from chickens infected with H9N2 have been shown to induce cross-reactivivity of cell-mediated immunity against the H7N2 isolate (Kapczynski et al., 2011). Such evidence suggests that H9N2-induced cellular immunity can provide cross-protection against strains of other subtypes of AIVs.

The change trends of CD4 and CD8 T cells in the PBMCs of H9N2-infected chickens have varied in different studies. Qiang et al. found that the proportion of CD4 T cells in PBMCs after H9N2 AIV infection was lower than that in the uninfected group, and there was a significant difference at 14 and 21 d post-infection (dpi). Meanwhile, the proportion of CD8 T cells in PBMCs was significantly lower only at 14 dpi, and the difference was not significant at any of the other time point (Qiang and Youxiang, 2011). However, other studies have reported no significant differences in the proportion of CD4 T cells in PBMCs after H9N2 AIV infection but observed a significant increase in the proportion of CD8 T cells at 3 and 5 dpi (Huang et al., 2012; Kwon et al., 2008). Moreover, Dai et al. found that although the trend of CD8 was also upregulated at 5 dpi, the proportion of CD4 cells was significantly upregulated after 5 dpi with H9N2 (Dai et al., 2021). Another study, however, reported no change in the proportion of CD4 and CD8 cells (Teng et al., 2006) similarly to our unpublished study. Although those differences may derive from differences in chicken breeds and MHC genotyping, we believe that H9N2 AIV infection in chickens may not cause significant changes in the proportion of CD4 and CD8 T cell in PBMCs, because the H9N2 virus is a LPAIV and cannot cause obvious clinical symptoms.

#### 3.1.2. Regulatory T (Treg) cells

Treg cells are a subset of CD4 T cells that regulate the immune system negatively by providing feedback mechanisms. They protect hosts from excessive immune responses during infection and maintain self-tolerance and mucosal tolerance (Workman et al., 2009). Treg cells were first described as CD4^+^CD25^+^ in mice (Sakaguchi et al., 1995), and later FOXP3 was identified as a critical regulator of Treg cells (Schubert et al., 2001), which is necessary for their feedback function. Mammalian Treg cells do not produce IL-2 (Shevach, 2009) and inhibit the proliferation of T cells by competing with them to bind to IL-2 *via* CD25, a high-affinity IL-2 receptor. This suppressive effect of Treg cells can be reversed by adding IL-2 *in vitro* (Scheffold et al., 2005; Selvaraj, 2013). These mechanisms have also been demonstrated in chicken (Shanmugasundaram and Selvaraj, 2011). *In vivo*, the immunosuppressive response of Treg cells can be reduced by adding anti-CD25 antibodies (Shanmugasundaram and Selvaraj, 2012; Shanmugasundaram and Selvaraj, 2013). Treg cells also express CTLA-4 which can competitively bind to and lower the levels of CD80/CD86 co-stimulatory molecules on APCs, thus inhibiting T cell activation. Moreover, Treg cells also produce various immune-suppressive cytokines, such as IL-10, TGF-β, and IL-35 (Tanaka and Sakaguchi, 2017). However, excessive activation of Treg cells can severely inhibit the immune function of T cells, B cells, NK cells, DCs, and macrophages by cytokines and cell-to-cell contact mechanisms (Sakaguchi et al., 2008), leading to chronic disease and poor defense against pathogens (Li et al., 2008). Treg cells not only affect immune response to self-antigens, but also participate in the immune response to viruses in almost every case studied (Belkaid and Tarbell, 2009; Rouse et al., 2006; Rudensky and Campbell, 2006).

It was previously thought that chicken did not have the FOXP3 gene and used CD4^+^CD25^+^ as Treg cells (Selvaraj, 2013; Shack et al., 2008). However, in 2013 researchers found FOXP3 in *Pseudopodoces humilis* using sequencing technology and bioinformatics (Qu et al., 2013). More recently, Burkhardt et al. successfully cloned chicken FOXP3 despite the highly GC-rich content of the chicken FOXP3 gene that makes it difficult to amplify. They found that FOXP3 is expressed on CD4^+^CD25^+^ in peripheral lymphoid organs such as the spleen and cecal tonsils, but not on CD4^+^CD25^+^ in the thymus, which correlates with the origin of chicken FOXP3^+^ T cells. They also identified a CD4^−^CD25^+^FOXP3^high^ subset of thymic lymphocytes, which suggests a distinct FOXP3 population in chicken compared with mammals (Burkhardt et al., 2022). Another study showed that CD25^+^, CD4^+^CD25^+^, and CD8^+^CD25^+^ cells in the spleen of chicken were significantly upregulated at 2 dpi with H9N2 but then decreased and stabilized (Teng et al., 2006). Other studies have shown that only CD8^+^CD25^+^ cells were significantly upregulated in the PBMCs of H9N2-infected chickens at 3 dpi and then decreased (Huang et al., 2012). Similar results have also been found in mice. Influenza A virus induced a strong FOXP3^+^CD4^+^ T cell response that was highly suppressive regarding CD4^+^ and CD8^+^ T cell effects *in vitro*. These Treg cells proliferated rapidly in response to influenza virus antigens (Betts et al., 2012). However, the effects of Treg-mediated suppression on infection *in vivo* remain uncertain. On one hand, Treg activity helps to control excessive immune responses induced by pathogens that could damage tissue (Workman et al., 2009). On the other hand, several studies have shown that Treg cells delay the clearance of virus from hosts and prolong the infection in mammals (Cabrera et al., 2004; Kinter et al., 2004; Stoop et al., 2005; Suvas et al., 2003). Moreover, this persistent infection-mediated immune response affects subsequent infections of repeated pathogens (Belkaid et al., 2002).

### 3.2. Antibody response and B cells

The humoral immune response is an important mechanism that enables hosts to maintain the homeostasis of the extracellular humoral microenvironment. In this process, antibodies produced by B cells are the mainly means of humoral immunity against infectious agents (Hua and Hou, 2013). There are two types of B cell-mediated immune responses: thymus-dependent antigens (TD-Ag) and thymus-independent antigens TI-Ag. Similar to T cells, B cell activation by TD-Ag needs three signals: an antigen stimulation signal, co-stimulatory molecules on the surface of Th cells and B cells, and cytokines, including IL-21 (Depoil et al., 2009; Kuchen et al., 2007). As APCs, B cells transmit antigens and costimulatory signals to T cells. Then T cells send a second signal to B cells by co-stimulatory molecules after receiving the antigen signals (Parker, 1993).

Researchers have demonstrated that the activation of chicken B cells is similar to that of mammals, in that both require an MHC class II-restricted interaction between the helper T cell and the responding B cell (Vainio et al., 1984). Chicken B cells can also form GCs, which have been shown to induce the proliferation of B cells (Yasuda et al., 1998) and immunoglobulin class switching (Arakawa et al., 1996; Yasuda et al., 2003), but evidence of whether they induce antibody affinity maturation is lacking (Ratcliffe and Härtle, 2022). Antibodies are an important line of defense against pathogen invasion, as well as an important indicator for evaluating the immune effect of vaccines (Nutt et al., 2015). Studies have revealed that natural IgM antibody, generated by innate-like B cells called ‘B-1 cells’, was present in mice not infected with influenza virus and played a role in early infection (Baumgarth et al., 2000; Choi et al., 2012; Savage et al., 2017). Subsequently, a part of B cells co-activated by influenza Ag and Th cells differentiate into plasma cells to secrete multiple antibodies (Lam and Baumgarth, 2019). Meanwhile, another part undergoes a germinal GC reaction that generates a broad repertoire of antibodies and B memory cells in preparation for the next exposure to the virus (Lam and Baumgarth, 2019). This interaction between T and B cells is critical for the development of a robust immune response. The antibodies produced by B cells can recognize and neutralize specific antigens, thereby preventing or limiting the spread of infection. Overall, the humoral immune response is a complex process involving multiple signaling pathways and cell types that work together to protect the body against infectious agents.

The bursa of Fabricius is unique in birds and generates primary B lymphocytes. It is populated by B cell precursors from about EID 8 to EID 14 (Houssaint et al., 1976; Le Douarin et al., 1975). Although it was initially suggested that H9N2 could inhibit humoral immunity in chickens leading to poor production of neutralizing antibodies (Xing et al., 2008), this may not be reliable because those chicken came from commercial egg farms infected with an unknown virus. In contrast, studies have clearly shown that H9N2 can effectively induce HI antibodies after infecting chickens (Dai et al., 2021). Other studies have predicted the B cell epitopes of H9N2 based on the development of peptide-based vaccines and revealed that the discontinuous epitopes of HA and HA are in amino acid residue positions 136–521 and 107–469, respectively (Ramamurthy et al., 2019). They also found potentially and highly immunogenic linear and conformational B-cell epitopes, which may support the development of a vaccine against AIVs for both humans and poultry.

### 3.3. Mucosal immune response

The respiratory mucosa is the first target of influenza virus attack, constituting the first line of defense against infection and effectively preventing the virus from entering (Ross and Herzberg, 2016). The protective immunity against respiratory virus infection involves a complex interaction between systemic and mucosal responses (Broadbent et al., 2015). The mucosal immune system in the upper respiratory tract plays a vital role in controlling influenza virus infection. It produces antiviral and immunomodulatory factors, including natural immunoglobulins, antimicrobial peptides, lectins, complement molecules and mucins, which respond first to influenza virus infection (Malik and Zhou, 2020). Then adaptive immune responses are activated to exert antiviral effects, among which antibodies play an important role, especially IgG and IgA. IgG antibodies mainly reduce viral pneumonia rather than prevent upper respiratory tract infections *via* the mucosa (Ito et al., 2003). Compared with IgG, IgA plays a more important role in preventing influenza virus infection in the upper respiratory tract (Suzuki et al., 2015) and clearing from infection with epithelial viruses (Brandtzaeg, 2007). In addition, influenza virus-induced respiratory mucosal IgA shows stronger cross-reactivity to different subtypes of virus strains than serum IgG (Asahi-Ozaki et al., 2004; Sano et al., 2018; Tamura et al., 1991). The blood-gas barrier of bird lungs is much thinner than that of mammals of equal weight, while the breathing surface is larger. These characteristics are considered to be factors that make pathogens more likely to cause pathological damage to bird lungs (Reese et al., 2006). The peripheral lymphatic system involved in the mucosal immune response of birds respiratory tract mainly includes: Harderian gland (HG), conjunctiva-associated lymphoid tissue (CALT), head-associated lymphoid tissue (HALT), and bronchus-associated lymphoid tissue (BALT) (Rumińska et al., 2008). BALT is a normal lymphoid structure in poultry that compensates for the lack of lymph node system (Fagerland and Arp, 1993). Fully developed BALT has been found in birds at 6 weeks of age and older, with almost every bronchus opening surrounded by lymphoid tissue (Fagerland and Arp, 1993). The Ig secreted by HG can enter the upper respiratory tract, providing localized protection (Dohms et al., 1981). Surgical removal of the HGs indicates that the main source of IgA in tears is HGs (Baba et al., 1990). The level of virus-specific IgA is correlated with the level of protection against ocular challenge (Gelb et al., 1998; Toro and Fernandez, 1994). Harderian glands play a role in the development of local immunity against various pathogens (Burns, 1979; Davelaar and Kouwenhoven, 1980; Survashe and Altken, 1977). Unfortunately, in-depth research on the chicken respiratory mucosal immune system was mainly concentrated in the last century, and has been stagnant for more than 20 years, and there is no recent research on how the mucosal immune system responds to avian influenza.

## Conclusions

Knowledge about the avian immune system has contributed greatly to our understanding of modern basic immunology, especially that of chicken. Studies on the immune system of chicken have pioneered an explanation for the two major systems of adaptive immunity: humoral immunity and cellular immunity (Davison, 2022). In an accidental experiment, Glick B discovered that the bursa of Fabricius is an essential organ for mediating antibody immune response (Glick, 1987). In the years that followed, mainstream immunologists began to take an interest in chicken lymphoid organs and concluded that the thymus controls cell-mediated immunity (Szenberg and Warner, 1962; Warner and Szenberg, 1962; Warner et al., 1962). In 1965, Cooper et al. proposed an initial description of the bursa of Fabricius and thymic lymphatic system of chicken (Cooper et al., 1965). Since then, the central concept of immunology, involving T-cell-mediated cellular immunity and B-cell-mediated humoral immunity, has been established. To commemorate the discovery of this special immune organ structure of birds and its contribution to immunology, the cells that mediate humoral immunity are named ‘bursa-derived lymphocytes’—that is B lymphocytes (Davison, 2022).

However, compared with studies on mammals, research on the immune system of chicken has been limited and lagging behind in recent decades. This is largely due to the following challenges: non-standardized methods of assessing avian cellular immunity; a lack of antibodies for chicken, including the unstable reproducibility of intracellular IFN-γ cytokine staining; and the lack of antibodies against markers for T cell activation and migration (Hao et al., 2021). Nevertheless, chicken have a population numbering in the tens of billions worldwide, they provide a vast pool of hosts for AIVs, which provides countless opportunities for avian influenza to multiply and mutate and, ultimately honing its ability to cross the host barrier from chickens to mammals and even humans. Among these viruses, the H9N2 virus is a major source of gene segments for a variety of influenza viruses that infect humans. Given these trends, it is crucial to control the prevalence of H9N2 in chicken, which requires a profound understanding of the immune system of chicken and its role in defending against H9N2.

In this review, we have discussed how innate and adaptive immune responses cooperate to control viral infection following H9N2 AIV infection in chicken. As is known, a host needs multiple immune responses to work together against H9N2. Although a single immune response can be pivotal, it is not enough to completely eliminate the virus. In past studies, more attention was paid to the importance of antibodies in protecting against H9N2 subtype AIVs. As a consequence, current understanding of the role of cellular immunity and innate immune responses in preventing H9N2 in chickens remains limited. Therefore, future research needs to explore the immune response of chickens to H9N2 from multiple perspectives and provide a theoretical basis for controlling its spread in chickens, or an improved strategy for constructing vaccines against it.

## References

[CIT0001] Abdolmaleki M, Yeap SK, Tan SW, Satharasinghe DA, Bello MB, Jahromi MZ, Bejo MH, Omar AR, Ideris A., 2018. Effects of Newcastle Disease Virus Infection on Chicken Intestinal Intraepithelial Natural Killer Cells. Front Immunol. 9:1386. doi: 10.3389/fimmu.2018.01386. 29973933 PMC6019501

[CIT0002] Ahad A Rabbani, et al. 2013. Serosurveillance to H9 and H7 Avian Influenza Virus among Poultry Workers in Punjab Province, Pakistan. Pakistan Veterinary Journal. 33(1):107–112.

[CIT0003] Akauliya M, Gautam A, Maharjan S, Park BK, Kim J, Kwon HJ. 2020. CD83 expression regulates antibody production in response to influenza A virus infection. Virol J. 17(1):194. doi: 10.1186/s12985-020-01465-0. 33302987 PMC7730749

[CIT0004] Alexander DJ. 2007. An overview of the epidemiology of avian influenza. Vaccine. 25(30):5637–5644. doi: 10.1016/j.vaccine.2006.10.051. 17126960

[CIT0005] Alizadeh E, Hosseini SM, Kheiri MT, Mazaheri V, Tabatabaeian M. 2009. Avian Influenza (H9N2) among poultry workers in Iran. Iranian Journal of Microbiology. 1(3):3–6.

[CIT0006] Alkie TN, Yitbarek A, Hodgins DC, Kulkarni RR, Taha-Abdelaziz K, Sharif S. 2019. Development of innate immunity in chicken embryos and newly hatched chicks: a disease control perspective. Avian Pathol. 48(4):288–310. doi: 10.1080/03079457.2019.1607966. 31063007

[CIT0007] Arakawa H, Furusawa S, Ekino S, Yamagishi H. 1996. Immunoglobulin gene hyperconversion ongoing in chicken splenic germinal centers. Embo J. 15(10):2540–2546. 8665861 PMC450186

[CIT0008] Arnon TI, Lev M, Katz G, Chernobrov Y, Porgador A, Mandelboim O. 2001. Recognition of viral hemagglutinins by NKp44 but not by NKp30. Eur J Immunol. 31(9):2680–2689. doi: 10.1002/1521-4141(200109)31:9<2680::AID-IMMU2680>3.0.CO;2-A. 11536166

[CIT0009] Asahi-Ozaki Y, Yoshikawa T, Iwakura Y, Suzuki Y, Tamura S-I, Kurata T, Sata T. 2004. Secretory IgA antibodies provide cross-protection against infection with different strains of influenza B virus. J Med Virol. 74(2):328–335., doi: 10.1002/jmv.20173. 15332283

[CIT0010] Baba T, Kawata T, Masumoto K, Kajikawa T. 1990. Role of the harderian gland in immunoglobulin A production in chicken lacrimal fluid. Res Vet Sci. 49(1):20–24. doi: 10.1016/S0034-5288(18)31039-7. 2116654

[CIT0011] Babior BM. 1992. The respiratory burst oxidase. Adv Enzymol Relat Areas Mol Biol. 65:49–95. 1570769 10.1002/9780470123119.ch2

[CIT0012] Babior BM. 2004. NADPH oxidase. Curr Opin Immunol. 16(1):42–47. doi: 10.1016/j.coi.2003.12.001. 14734109

[CIT0013] Bang BG, Bang FB. 1968. Localized lymphoid tissues and plasma cells in paraocular and paranasal organ systems in chickens. The American Journal of Pathology. 53:735–751. 5693341 PMC2013518

[CIT0014] Baumgarth N, Herman OC, Jager GC, Brown LE, Herzenberg LA, Chen J. 2000. B-1 and B-2 cell-derived immunoglobulin M antibodies are nonredundant components of the protective response to influenza virus infection. J Exp Med. 192(2):271–280. doi: 10.1084/jem.192.2.271. 10899913 PMC2193249

[CIT0015] Belkaid Y, Piccirillo CA, Mendez S, Shevach EM, Sacks DL. 2002. CD4 + CD25+ regulatory T cells control Leishmania major persistence and immunity. Nature. 420(6915):502–507. doi: 10.1038/nature01152. 12466842

[CIT0016] Belkaid Y, Tarbell K. 2009. Regulatory T cells in the control of host-microorganism interactions (*). Annu Rev Immunol. 27:551–589. doi: 10.1146/annurev.immunol.021908.132723. 19302048

[CIT0017] Betts RJ, Prabhu N, Ho AWS, Lew FC, Hutchinson PE, Rotzschke O, Macary PA, Kemeny DM. 2012. Influenza A virus infection results in a robust, antigen-responsive, and widely disseminated Foxp3+ regulatory T cell response. J Virol. 86(5):2817–2825., doi: 10.1128/JVI.05685-11. 22205730 PMC3302292

[CIT0018] Bi Y, Li J, Li S, Fu G, Jin T, Zhang C, Yang Y, Ma Z, Tian W, Li J, et al. 2020. Dominant subtype switch in avian influenza viruses during 2016-2019 in China. Nat Commun. 11(1):5909. doi: 10.1038/s41467-020-19671-3. 33219213 PMC7679419

[CIT0019] Blair PJ, Putnam SD, Krueger WS, Chum C, Wierzba TF, Heil GL, Yasuda CY, Williams M, Kasper MR, Friary JA, et al. 2013. Evidence for avian H9N2 influenza virus infections among rural villagers in Cambodia. J Infect Public Health. 6(2):69–79. doi: 10.1016/j.jiph.2012.11.005. 23537819 PMC3612269

[CIT0020] Brandtzaeg P. 2007. Induction of secretory immunity and memory at mucosal surfaces. Vaccine. 25(30):5467–5484. doi: 10.1016/j.vaccine.2006.12.001. 17227687

[CIT0021] Broadbent AJ, Boonnak K, Subbarao K. 2015. Chapter 59—Respiratory virus vaccines. Mucosal Immunology. (Fourth Edition). 1:1129–1170.

[CIT0022] Brownlie R, Allan B. 2011. Avian toll-like receptors. Cell Tissue Res. 343(1):121–130. doi: 10.1007/s00441-010-1026-0. 20809414

[CIT0023] Burkhardt NB, Elleder D, Schusser B, Krchlíková V, Göbel TW, Härtle S, Kaspers B. 2022. The Discovery of Chicken Foxp3 Demands Redefinition of Avian Regulatory T Cells. J Immunol. 208(5):1128–1138., doi: 10.4049/jimmunol.2000301. 35173035

[CIT0024] Burns RB. 1979. Histological and immunological studies on the fowl lacrimal gland following surgical excision of Harder’s gland. Res Vet Sci. 27(1):69–75. doi: 10.1016/S0034-5288(18)32861-3. 116336

[CIT0025] Cabrera R, Tu Z, Xu Y, Firpi RJ, Rosen HR, Liu C, Nelson DR. 2004. An immunomodulatory role for CD4(+)CD25(+) regulatory T lymphocytes in hepatitis C virus infection. Hepatology. 40(5):1062–1071., doi: 10.1002/hep.20454. 15486925

[CIT0026] Chen H, Yuan H, Gao R, Zhang J, Wang D, Xiong Y, Fan G, Yang F, Li X, Zhou J, et al. 2014. Clinical and epidemiological characteristics of a fatal case of avian influenza A H10N8 virus infection: a descriptive study. Lancet. 383(9918):714–721. doi: 10.1016/S0140-6736(14)60111-2. 24507376

[CIT0027] Cheng Y, Sun Y, Wang H, Yan Y, Ding C, Sun J. 2015. Chicken STING Mediates Activation of the IFN Gene Independently of the RIG-I Gene. J Immunol. 195(8):3922–3936. doi: 10.4049/jimmunol.1500638. 26392466

[CIT0028] Choi YS, Dieter JA, Rothaeusler K, Luo Z, Baumgarth N. 2012. B-1 cells in the bone marrow are a significant source of natural IgM. Eur J Immunol. 42(1):120–129. doi: 10.1002/eji.201141890. 22009734 PMC3426357

[CIT0029] Chothe SK, Nissly RH, Lim L, Bhushan G, Bird I, Radzio-Basu J, Jayarao BM, Kuchipudi SV., 2020. NLRC5 Serves as a Pro-viral Factor During Influenza Virus Infection in Chicken Macrophages. Front Cell Infect Microbiol. 10:230. doi: 10.3389/fcimb.2020.00230. 32509599 PMC7248199

[CIT0030] Chu J, Guo Y, Xu G, Zhang Q, Zuo Z, Li Q, Wang Y, He C. 2020. Chlamydia psittaci Triggers the Invasion of H9N2 Avian Influenza Virus by Impairing the Functions of Chicken Macrophages. Animals (Basel. 10(4):722., doi: 10.3390/ani10040722. 32326284 PMC7222846

[CIT0031] Cline TD, Beck D, Bianchini E. 2017. Influenza virus replication in macrophages: balancing protection and pathogenesis. J Gen Virol. 98(10):2401–2412. doi: 10.1099/jgv.0.000922. 28884667 PMC5725990

[CIT0032] Coman A, Maftei DN, Krueger WS, Heil GL, Friary JA, Chereches RM, Sirlincan E, Bria P, Dragnea C, Kasler I, et al. 2013. Serological evidence for avian H9N2 influenza virus infections among Romanian agriculture workers. J Infect Public Health. 6(6):438–447. doi: 10.1016/j.jiph.2013.05.003. 23999337

[CIT0033] Cong J, Wei H. 2019. Natural Killer Cells in the Lungs. Front Immunol. 10:1416. doi: 10.3389/fimmu.2019.01416. 31293580 PMC6603080

[CIT0034] Cong YL, Pu J, Liu QF, Wang S, Zhang GZ, Zhang XL, Fan WX, Brown EG, Liu JH. 2007. Antigenic and genetic characterization of H9N2 swine influenza viruses in China. J Gen Virol. 88(Pt 7):2035–2041., doi: 10.1099/vir.0.82783-0. 17554038

[CIT0035] Cooper MD, Peterson RDA, Good RA. 1965. Delineation of the Thymic and Bursal Lymphoid Systems in the Chicken. Nature. 205:143–146. doi: 10.1038/205143a0. 14276257

[CIT0036] Curtsinger JM, Mescher MF. 2010. Inflammatory cytokines as a third signal for T cell activation. Curr Opin Immunol. 22(3):333–340. doi: 10.1016/j.coi.2010.02.013. 20363604 PMC2891062

[CIT0037] Dai, Manman, Li, Shibing, Sun, Hui, Zhao, Li, Liao, Jiayu, Xu, Chenggang, Liao, Ming, Keyi Shi, Deshui Yu, 2021. Comparative analysis of key immune protection factors in H9N2 avian influenza viruses infected and immunized specific pathogen-free chicken. Poult Sci, 1 100:39–46 doi: 10.1016/j.psj.2020.09.080. 33357705 PMC7772655

[CIT0038] Dalpke A, Frank J, Peter M, Heeg K. 2006. Activation of toll-like receptor 9 by DNA from different bacterial species. Infect Immun. 74(2):940–946. doi: 10.1128/IAI.74.2.940-946.2006. 16428738 PMC1360326

[CIT0039] Davelaar FG, Kouwenhoven B. 1980. Effect of the removal of the Harderian gland in 1-day-old chicks on immunity following IB vaccination. Avian Pathol. 9(4):489–497. doi: 10.1080/03079458008418436. 18770290

[CIT0040] Davison F. 2022. The importance of the avian immune system and its unique features. In Avian immunology. UK: Elsevier. pp. 1–9.

[CIT0041] Degen WG, van Daal N, van Zuilekom HI, Burnside J, Schijns VE. 2004. Identification and molecular cloning of functional chicken IL-12. J Immunol. 172(7):4371–4380. doi: 10.4049/jimmunol.172.7.4371. 15034052

[CIT0042] Depoil D, Weber M, Treanor B, Fleire SJ, Carrasco YR, Harwood NE, Batista FD. 2009. Early events of B cell activation by antigen. Sci Signal. 2(63):pt1., doi: 10.1126/scisignal.263pt1. 19318623

[CIT0043] Dohms JE, Lee KP, Rosenberger JK. 1981. Plasma cell changes in the gland of Harder following infectious bursal disease virus infection of the chicken. Avian Dis. 25(3):683–695. doi: 10.2307/1589999. 6274296

[CIT0044] Fagerland JA, Arp LH. 1993. Distribution and quantitation of plasma cells, T lymphocyte subsets, and B lymphocytes in bronchus-associated lymphoid tissue of chickens: age-related differences. Regional Immunology. 5:28–36. 8347468

[CIT0045] Fagerland JA, Arp LH. 1993. Structure and development of bronchus-associated lymphoid tissue in conventionally reared broiler chickens. Avian Dis. 37(1):10–18. doi: 10.2307/1591451. 8452486

[CIT0046] Fioretti A, Calabria M, Piccirillo A, Menna L. 1999. The epidemiological situation of avian influenza in Italy during 1998. In Proc. Joint Fifth Annual Meetings of the National Newcastle Disease and Avian Influenza Laboratories of Countries of the European Union, Vienna. Citeseer. pp p. 20–22.

[CIT0047] Gelb J, Jr., Nix WA, Gellman SD. 1998. Infectious bronchitis virus antibodies in tears and their relationship to immunity. Avian Dis. 42(2):364–374. doi: 10.2307/1592487. 9645328

[CIT0048] Glick B. 1987. How it all began: the continuing story of the bursa of Fabricius.

[CIT0049] Göbel TW, Chen CL, Shrimpf J, Grossi CE, Bernot A, Bucy RP, Auffray C, Cooper MD. 1994. Characterization of avian natural killer cells and their intracellular CD3 protein complex. Eur J Immunol. 24(7):1685–1691., doi: 10.1002/eji.1830240734. 8026528

[CIT0050] Guan Y, Shortridge KF, Krauss S, Webster RG. 1999. Molecular characterization of H9N2 influenza viruses: were they the donors of the "internal" genes of H5N1 viruses in Hong Kong? Proc Natl Acad Sci U S A. 96(16):9363–9367. doi: 10.1073/pnas.96.16.9363. 10430948 PMC17788

[CIT0051] Guo J, Wang Y, Zhao C, Gao X, Zhang Y, Li J, Wang M, Zhang H, Liu W, Wang C, et al. 2021. Molecular characterization, receptor binding property, and replication in chickens and mice of H9N2 avian influenza viruses isolated from chickens, peafowls, and wild birds in eastern China. Emerg Microbes Infect. 10(1):2098–2112. doi: 10.1080/22221751.2021.1999778. 34709136 PMC8592596

[CIT0052] Guo P, Thomas JD, Bruce MP, Hinton TM, Bean AG, Lowenthal JW. 2013. The chicken TH1 response: potential therapeutic applications of ChIFN-γ. Dev Comp Immunol. 41(3):389–396. doi: 10.1016/j.dci.2013.05.009. 23707786

[CIT0053] Guo Y, Li J, Cheng X. 1999. Discovery of men infected by avian influenza A (H9N2) virus]. Zhonghua shi yan he lin chuang bing du xue za zhi = Zhonghua shiyan he linchuang bingduxue zazhi =. Chinese Journal of Experimental and Clinical Virology. 13:105–108. 12569771

[CIT0054] Gutcher I, Becher B. 2007. APC-derived cytokines and T cell polarization in autoimmune inflammation. J Clin Invest. 117(5):1119–1127. doi: 10.1172/JCI31720. 17476341 PMC1857272

[CIT0055] Hao X, Zhang F, Yang Y, Shang S. 2021. The Evaluation of Cellular Immunity to Avian Viral Diseases: methods, Applications, and Challenges. Front Microbiol. 12:794514. doi: 10.3389/fmicb.2021.794514. 34950125 PMC8689181

[CIT0056] Hayashi T, Watanabe C, Suzuki Y, Tanikawa T, Uchida Y, Saito T. 2014. Chicken MDA5 senses short double-stranded RNA with implications for antiviral response against avian influenza viruses in chicken. J Innate Immun. 6(1):58–71. doi: 10.1159/000351583. 23860388 PMC6741575

[CIT0057] He H, Genovese KJ, Kogut MH. 2011. Modulation of chicken macrophage effector function by T(H)1/T(H)2 cytokines. Cytokine. 53(3):363–369. doi: 10.1016/j.cyto.2010.12.009. 21208811

[CIT0058] He W-T, Hou X, Zhao J, Sun J, He H, Si W, Wang J, Jiang Z, Yan Z, Xing G, et al. 2022. Virome characterization of game animals in China reveals a spectrum of emerging pathogens. Cell. 185(7):1117–1129.e8. e1118 doi: 10.1016/j.cell.2022.02.014. 35298912 PMC9942426

[CIT0059] He Z, Ma Y, Wu D, Feng W, Xiao J. 2021. Protective effects of the NLRP3 inflammasome against infectious bursal disease virus replication in DF-1 cells. Arch Virol. 166(7):1943–1950. doi: 10.1007/s00705-021-05099-7. 33982180

[CIT0060] Ho JW, Hershkovitz O, Peiris M, Zilka A, Bar-Ilan A, Nal B, Chu K, Kudelko M, Kam YW, Achdout H, et al. 2008. H5-type influenza virus hemagglutinin is functionally recognized by the natural killer-activating receptor NKp44. J Virol. 82(4):2028–2032. doi: 10.1128/JVI.02065-07. 18077718 PMC2258730

[CIT0061] Homme PJ, Easterday BC. 1970. Avian influenza virus infections. I. Characteristics of influenza A-turkey-Wisconsin-1966 virus. Avian Dis. 14(1):66–74. doi: 10.2307/1588557. 4314007

[CIT0062] Houssaint E, Belo M, Le Douarin NM. 1976. Investigations on cell lineage and tissue interactions in the developing bursa of Fabricius through interspecific chimeras. Dev Biol. 53(2):250–264. doi: 10.1016/0012-1606(76)90227-x. 992209

[CIT0063] Hua Z, Hou B. 2013. TLR signaling in B-cell development and activation. Cell Mol Immunol. 10(2):103–106. doi: 10.1038/cmi.2012.61. 23241902 PMC4003046

[CIT0064] Huang Z, Fang D, Lv P, Bian X, Ruan X, Yan Y, Zhou J. 2012. Differential cellular immune responses between chickens and ducks to H9N2 avian influenza virus infection. Vet Immunol Immunopathol. 150(3-4):169–180., doi: 10.1016/j.vetimm.2012.09.010. 23063347

[CIT0065] Ito R, Ozaki YA, Yoshikawa T, Hasegawa H, Sato Y, Suzuki Y, Inoue R, Morishima T, Kondo N, Sata T, et al. 2003. Roles of anti-hemagglutinin IgA and IgG antibodies in different sites of the respiratory tract of vaccinated mice in preventing lethal influenza pneumonia. Vaccine. 21(19-20):2362–2371. doi: 10.1016/s0264-410x(03)00078-1. 12744867

[CIT0066] Jansen CA, de Geus ED, van Haarlem DA, van de Haar PM, Löndt BZ, Graham SP, Göbel TW, van Eden W, Brookes SM, Vervelde L, et al. 2013. Differential lung NK cell responses in avian influenza virus infected chickens correlate with pathogenicity. Sci Rep. 3:2478. doi: 10.1038/srep02478. 23963354 PMC3748423

[CIT0067] Jeurissen SH, Janse EM. 1989. Distribution and function of non-lymphoid cells in liver and spleen of embryonic and adult chickens. Prog Clin Biol Res. 307:149–157. 2678136

[CIT0068] Kägi D, Ledermann B, Bürki K, Seiler P, Odermatt B, Olsen KJ, Podack ER, Zinkernagel RM, Hengartner H. 1994. Cytotoxicity mediated by T cells and natural killer cells is greatly impaired in perforin-deficient mice. Nature. 369(6475):31–37., doi: 10.1038/369031a0. 8164737

[CIT0069] Kalaiyarasu S, Kumar M, Senthil Kumar D, Bhatia S, Dash SK, Bhat S, Khetan RK, Nagarajan S. 2016. Highly pathogenic avian influenza H5N1 virus induces cytokine dysregulation with suppressed maturation of chicken monocyte-derived dendritic cells. Microbiol Immunol. 60(10):687–693., doi: 10.1111/1348-0421.12443. 27730669

[CIT0070] Kapczynski DR, Liljebjelke K, Kulkarni G, Hunt H, Jiang HJ, Petkov D. 2011. Cross reactive cellular immune responses in chickens previously exposed to low pathogenic avian influenza. BMC Proc. 5 Suppl 4(Suppl 4):S13. doi: 10.1186/1753-6561-5-S4-S13. PMC3108207 21645292

[CIT0071] Kinter AL, Hennessey M, Bell A, Kern S, Lin Y, Daucher M, Planta M, McGlaughlin M, Jackson R, Ziegler SF, et al. 2004. CD25(+)CD4(+) regulatory T cells from the peripheral blood of asymptomatic HIV-infected individuals regulate CD4(+) and CD8(+) HIV-specific T cell immune responses in vitro and are associated with favorable clinical markers of disease status. J Exp Med. 200(3):331–343. doi: 10.1084/jem.20032069. 15280419 PMC2211981

[CIT0072] Ku KB, Park EH, Yum J, Kim HM, Kang YM, Kim JC, Kim JA, Kim HS, Seo SH., 2014. Transmissibility of novel H7N9 and H9N2 avian influenza viruses between chickens and ferrets. Virology. 450-451:316–323. doi: 10.1016/j.virol.2013.12.022. 24503095

[CIT0073] Kuchen S, Robbins R, Sims GP, Sheng C, Phillips TM, Lipsky PE, Ettinger R. 2007. Essential role of IL-21 in B cell activation, expansion, and plasma cell generation during CD4+ T cell-B cell collaboration. J Immunol. 179(9):5886–5896., doi: 10.4049/jimmunol.179.9.5886. 17947662

[CIT0074] Kwon J-S, Lee H-J, Lee D-H, Lee Y-J, Mo I-P, Nahm S-S, Kim M-J, Lee J-B, Park S-Y, Choi I-S, et al. 2008. Immune responses and pathogenesis in immunocompromised chickens in response to infection with the H9N2 low pathogenic avian influenza virus. Virus Res. 133(2):187–194. doi: 10.1016/j.virusres.2007.12.019. 18276028

[CIT0075] Lam JH, Baumgarth N. 2019. The Multifaceted B Cell Response to Influenza Virus. J Immunol. 202(2):351–359. doi: 10.4049/jimmunol.1801208. 30617116 PMC6327962

[CIT0076] Lam TT-Y, Wang J, Shen Y, Zhou B, Duan L, Cheung C-L, Ma C, Lycett SJ, Leung CY-H, Chen X, et al. 2013. The genesis and source of the H7N9 influenza viruses causing human infections in China. Nature. 502(7470):241–244. doi: 10.1038/nature12515. 23965623 PMC3801098

[CIT0077] Le Douarin NM, Houssaint E, Jotereau FV, Belo M. 1975. Origin of hemopoietic stem cells in embryonic bursa of Fabricius and bone marrow studied through interspecific chimeras. Proceedings of the National Academy of Sciences of the United States of America 72:p. 2701–2705. doi: 10.1073/pnas.72.7.2701. PMC432838 1101262

[CIT0078] Lee SB, Park YH, Chungu K, Woo SJ, Han ST, Choi HJ, Rengaraj D, Han JY., 2020. Targeted Knockout of MDA5 and TLR3 in the DF-1 Chicken Fibroblast Cell Line Impairs Innate Immune Response Against RNA Ligands. Front Immunol. 11:678. doi: 10.3389/fimmu.2020.00678. 32425931 PMC7204606

[CIT0079] Lee SH, Lillehoj HS, Jang SI, Lee KW, Baldwin C, Tompkins D, Wagner B, Del Cacho E, Lillehoj EP, Hong YH, et al. 2012. Development and characterization of mouse monoclonal antibodies reactive with chicken CD83. Vet Immunol Immunopathol. 145(1-2):527–533. doi: 10.1016/j.vetimm.2011.11.020. 22197010

[CIT0080] Li S, Gowans EJ, Chougnet C, Plebanski M, Dittmer U. 2008. Natural regulatory T cells and persistent viral infection. J Virol. 82(1):21–30. doi: 10.1128/JVI.01768-07. 17855537 PMC2224364

[CIT0081] Li S, Yang J, Zhu Y, Ji X, Wang K, Jiang S, Luo J, Wang H, Zheng W, Chen N, et al. 2020. Chicken DNA Sensing cGAS-STING Signal Pathway Mediates Broad Spectrum Antiviral Functions. Vaccines (Basel. 8(3):369. doi: 10.3390/vaccines8030369. 32660114 PMC7563795

[CIT0082] Li X, Tian B, Jianfang Z, Yongkun C, Xiaodan L, Wenfei Z, Yan L, Jing T, Junfeng G, Tao C, et al. 2017. A comprehensive retrospective study of the seroprevalence of H9N2 avian influenza viruses in occupationally exposed populations in China. PLoS One. 12(6):e0178328. doi: 10.1371/journal.pone.0178328. 28575073 PMC5456037

[CIT0083] Lin J, Yin Y, Y, Qin T, Zhu LQ, Yu QH, Yang Q. 2014. Enhanced immune response of BMDCs pulsed with H9N2 AIV and CpG. Vaccine. 32(50):6783–6790. doi: 10.1016/j.vaccine.2014.10.013. 25454862

[CIT0084] Liniger M, Summerfield A, Zimmer G, McCullough KC, Ruggli N. 2012. Chicken cells sense influenza A virus infection through MDA5 and CARDIF signaling involving LGP2. J Virol. 86(2):705–717. doi: 10.1128/JVI.00742-11. 22072756 PMC3255855

[CIT0085] Liu J, Zhang X, Cheng Y, Cao X. 2021. Dendritic cell migration in inflammation and immunity. Cell Mol Immunol. 18(11):2461–2471. doi: 10.1038/s41423-021-00726-4. 34302064 PMC8298985

[CIT0086] Liu Q, Yang J, Huang X, Liu Y, Han K, Zhao D, Zhang L, Li Y., 2020. Global gene expression analysis data of chicken dendritic cells infected with H9N2 avian influenza virus. Data Brief. 30:105430. doi: 10.1016/j.dib.2020.105430. 32300615 PMC7152653

[CIT0087] Liu Q, Yang J, Huang X, Liu Y, Han K, Zhao D, Zhang L, Li Y., 2020. Transcriptomic profile of chicken bone marrow-derive dendritic cells in response to H9N2 avian influenza A virus. Vet Immunol Immunopathol. 220:109992. doi: 10.1016/j.vetimm.2019.109992. 31846798

[CIT0088] Lowenthal JW, Connick TE, McWaters PG, York JJ. 1994. Development of T cell immune responsiveness in the chicken. Immunol Cell Biol. 72(2):115–122. doi: 10.1038/icb.1994.18. 8200687

[CIT0089] Malik G, Zhou Y. 2020. Innate Immune Sensing of Influenza A Virus. Viruses. 12(7):755. doi: 10.3390/v12070755. 32674269 PMC7411791

[CIT0090] Mandelboim O, Lieberman N, Lev M, Paul L, Arnon TI, Bushkin Y, Davis DM, Strominger JL, Yewdell JW, Porgador A, et al. 2001. Recognition of haemagglutinins on virus-infected cells by NKp46 activates lysis by human NK cells. Nature. 409(6823):1055–1060. doi: 10.1038/35059110. 11234016

[CIT0091] McMichael AJ, Gotch FM, Noble GR, Beare PA. 1983. Cytotoxic T-cell immunity to influenza. N Engl J Med. 309(1):13–17. doi: 10.1056/NEJM198307073090103. 6602294

[CIT0092] Meijerink N, van Haarlem DA, Velkers FC, Stegeman AJ, Rutten V, Jansen CA. 2021. Analysis of chicken intestinal natural killer cells, a major IEL subset during embryonic and early life. Dev Comp Immunol. 114:103857. doi: 10.1016/j.dci.2020.103857. 32891731

[CIT0093] Mock DJ, Domurat F, Roberts NJ, Jr., Walsh EE, Licht MR, Keng P. 1987. Macrophages are required for influenza virus infection of human lymphocytes. J Clin Invest. 79(2):620–624. doi: 10.1172/JCI112856. 3805284 PMC424144

[CIT0094] Murray PJ, Allen JE, Biswas SK, Fisher EA, Gilroy DW, Goerdt S, Gordon S, Hamilton JA, Ivashkiv LB, Lawrence T, et al. 2014. Macrophage activation and polarization: nomenclature and experimental guidelines. Immunity. 41(1):14–20. doi: 10.1016/j.immuni.2014.06.008. 25035950 PMC4123412

[CIT0095] Nang NT, Lee JS, Song BM, Kang YM, Kim HS, Seo SH. 2011. Induction of inflammatory cytokines and Toll-like receptors in chickens infected with avian H9N2 influenza virus. Vet Res. 42(1):64. doi: 10.1186/1297-9716-42-64. 21592354 PMC3114738

[CIT0096] Niu Q, Cheng Y, Wang H, Yan Y, Sun J. 2019. Chicken DDX3X Activates IFN-β via the chSTING-chIRF7-IFN-β Signaling Axis. Front Immunol. 10:822. doi: 10.3389/fimmu.2019.00822. 31057547 PMC6478769

[CIT0097] Nutt SL, Hodgkin PD, Tarlinton DM, Corcoran LM. 2015. The generation of antibody-secreting plasma cells. Nat Rev Immunol. 15(3):160–171. doi: 10.1038/nri3795. 25698678

[CIT0098] Okoye J, Eze D, Krueger WS, Heil GL, Friary JA, Gray GC. 2013. Serologic evidence of avian influenza virus infections among Nigerian agricultural workers. J Med Virol. 85(4):670–676. doi: 10.1002/jmv.23520. 23400898

[CIT0099] Palmquist JM, Khatri M, Cha RM, Goddeeris BM, Walcheck B, Sharma JM. 2006. In vivo activation of chicken macrophages by infectious bursal disease virus. Viral Immunol. 19(2):305–315. doi: 10.1089/vim.2006.19.305. 16817773

[CIT0100] Paoliello-Paschoalato AB, Oliveira SH, Cunha FQ. 2005. Interleukin 4 induces the expression of inducible nitric oxide synthase in eosinophils. Cytokine. 30(3):116–124. doi: 10.1016/j.cyto.2005.01.001. 15826818

[CIT0101] Parker DC. 1993. T cell-dependent B cell activation. Annu Rev Immunol. 11:331–360. doi: 10.1146/annurev.iy.11.040193.001555. 8476565

[CIT0102] Pawar SD, Tandale BV, Raut CG, Parkhi SS, Barde TD, Gurav YK, Kode SS, Mishra AC. 2012. Avian influenza H9N2 seroprevalence among poultry workers in Pune, India, 2010. PLoS One. 7(5):e36374., doi: 10.1371/journal.pone.0036374. 22623954 PMC3356154

[CIT0103] Qiang F, Youxiang D. 2011. The effects of H9N2 influenza A on the immune system of broiler chickens in the Shandong Province. Transbound Emerg Dis. 58(2):145–151. doi: 10.1111/j.1865-1682.2010.01192.x. 21205254

[CIT0104] Qu Y, Zhao H, Han N, Zhou G, Song G, Gao B, Tian S, Zhang J, Zhang R, Meng X, et al. 2013. Ground tit genome reveals avian adaptation to living at high altitudes in the Tibetan plateau. Nat Commun. 4:2071. doi: 10.1038/ncomms3071. 23817352

[CIT0105] Quan C, Wang Q, Zhang J, Zhao M, Dai Q, Huang T, Zhang Z, Mao S, Nie Y, Liu J, et al. 2019. Avian Influenza A Viruses among Occupationally Exposed Populations, China, 2014-2016. Emerg Infect Dis. 25(12):2215–2225. doi: 10.3201/eid2512.190261. 31742536 PMC6874249

[CIT0106] Qureshi MA. 2003. Avian macrophage and immune response: an overview. Poult Sci. 82(5):691–698. doi: 10.1093/ps/82.5.691. 12762389 PMC7194945

[CIT0107] RahimiRad S, Alizadeh A, Alizadeh E, Hosseini SM. 2016. The avian influenza H9N2 at avian-human interface: a possible risk for the future pandemics. J Res Med Sci. 21:51. doi: 10.4103/1735-1995.187253. 28083072 PMC5216463

[CIT0108] Ramamurthy M, Sankar S, Abraham AM, Nandagopal B, Sridharan G. 2019. B cell epitopes in the intrinsically disordered regions of neuraminidase and hemagglutinin proteins of H5N1 and H9N2 avian influenza viruses for peptide-based vaccine development. J Cell Biochem. 120(10):17534–17544. doi: 10.1002/jcb.29017. 31111560

[CIT0109] Ratcliffe MJ, Härtle S. 2022. B cells, the bursa of Fabricius, and the generation of antibody repertoires. In Avian immunology. Canada: Elsevier. pp. 71–99.

[CIT0110] Reese S, Dalamani G, Kaspers B. 2006. The avian lung-associated immune system: a review. Vet Res. 37(3):311–324. doi: 10.1051/vetres:2006003. 16611550

[CIT0111] Rogers SL, Viertlboeck BC, Göbel TW, Kaufman J. 2008. Avian NK activities, cells and receptors. Semin Immunol. 20(6):353–360. doi: 10.1016/j.smim.2008.09.005. 18948017

[CIT0112] Ross KF, Herzberg MC. 2016. Autonomous immunity in mucosal epithelial cells: fortifying the barrier against infection. Microbes Infect. 18(6):387–398. doi: 10.1016/j.micinf.2016.03.008. 27005450 PMC4884473

[CIT0113] Rouse BT, Sarangi PP, Suvas S. 2006. Regulatory T cells in virus infections. Immunol Rev. 212:272–286. doi: 10.1111/j.0105-2896.2006.00412.x. 16903920

[CIT0114] Rudensky AY, Campbell DJ. 2006. In vivo sites and cellular mechanisms of T reg cell-mediated suppression. J Exp Med. 203(3):489–492. doi: 10.1084/jem.20060214. 16533888 PMC2118229

[CIT0115] Rumińska E, Koncicki A, Stenzel T. 2008. Structure and function of the avian immune system in birds. Medycyna Weterynaryjna. 64:265–268.

[CIT0116] Sakaguchi S, Sakaguchi N, Asano M, Itoh M, Toda M. 1995. Immunologic self-tolerance maintained by activated T cells expressing IL-2 receptor alpha-chains (CD25). Breakdown of a single mechanism of self-tolerance causes various autoimmune diseases. J Immunol. 155(3):1151–1164. 7636184

[CIT0117] Sakaguchi S, Yamaguchi T, Nomura T, Ono M. 2008. Regulatory T cells and immune tolerance. Cell. 133(5):775–787. doi: 10.1016/j.cell.2008.05.009. 18510923

[CIT0118] Sano K, Ainai A, Suzuki T, Hasegawa H. 2018. Intranasal inactivated influenza vaccines for the prevention of seasonal influenza epidemics. Expert Rev Vaccines. 17(8):687–696. doi: 10.1080/14760584.2018.1507743. 30092690

[CIT0119] Savage HP, Yenson VM, Sawhney SS, Mousseau BJ, Lund FE, Baumgarth N. 2017. Blimp-1-dependent and -independent natural antibody production by B-1 and B-1-derived plasma cells. J Exp Med. 214(9):2777–2794. doi: 10.1084/jem.20161122. 28698287 PMC5584113

[CIT0120] Schat KA, Kaspers B, Kaiser P. 2014. Avian immunology. Second edition. ed. Elsevier: academic Press, Amsterdam; Boston

[CIT0121] Scheffold A, Hühn J, Höfer T. 2005. Regulation of CD4 + CD25+ regulatory T cell activity: it takes (IL-)two to tango. Eur J Immunol. 35(5):1336–1341. doi: 10.1002/eji.200425887. 15827965

[CIT0122] Schneider K, Puehler F, Baeuerle D, Elvers S, Staeheli P, Kaspers B, Weining KC. 2000. cDNA cloning of biologically active chicken interleukin-18. J Interferon Cytokine Res. 20(10):879–883., doi: 10.1089/10799900050163244. 11054275

[CIT0123] Schubert LA, Jeffery E, Zhang Y, Ramsdell F, Ziegler SF. 2001. Scurfin (FOXP3) acts as a repressor of transcription and regulates T cell activation. J Biol Chem. 276(40):37672–37679. doi: 10.1074/jbc.M104521200. 11483607

[CIT0124] Selvaraj RK. 2013. Avian CD4(+)CD25(+) regulatory T cells: properties and therapeutic applications. Dev Comp Immunol. 41(3):397–402. doi: 10.1016/j.dci.2013.04.018. 23665004

[CIT0125] Seo SH, Webster RG. 2001. Cross-reactive, cell-mediated immunity and protection of chickens from lethal H5N1 influenza virus infection in Hong Kong poultry markets. J Virol. 75(6):2516–2525. doi: 10.1128/JVI.75.6.2516-2525.2001. 11222674 PMC115873

[CIT0126] Seto F. 1981. Early development of the avian immune system. Poult Sci. 60(9):1981–1995. doi: 10.3382/ps.0601981. 6976568

[CIT0127] Shack LA, Buza JJ, Burgess SC. 2008. The neoplastically transformed (CD30hi) Marek’s disease lymphoma cell phenotype most closely resembles T-regulatory cells. Cancer Immunol Immunother. 57(8):1253–1262. doi: 10.1007/s00262-008-0460-2. 18256827 PMC11030954

[CIT0128] Shanmugasundaram R, Selvaraj RK. 2011. Regulatory T cell properties of chicken CD4 + CD25+ cells. J Immunol. 186(4):1997–2002. doi: 10.4049/jimmunol.1002040. 21242520

[CIT0129] Shanmugasundaram R, Selvaraj RK. 2012. Effects of in vivo injection of anti-chicken CD25 monoclonal antibody on regulatory T cell depletion and CD4 + CD25- T cell properties in chickens. Dev Comp Immunol. 36(3):578–583. doi: 10.1016/j.dci.2011.09.015. 22004798

[CIT0130] Shanmugasundaram R, Selvaraj RK. 2013. In ovo injection of anti-chicken CD25 monoclonal antibodies depletes CD4 + CD25+ T cells in chickens. Poult Sci. 92(1):138–142. doi: 10.3382/ps.2012-02593. 23243240

[CIT0131] Shen Y-Y, Ke C-W, Li Q, Yuan R-Y, Xiang D, Jia W-X, Yu Y-D, Liu L, Huang C, Qi W-B, et al. 2016. Novel Reassortant Avian Influenza A(H5N6) Viruses in Humans, Guangdong, China, 2015. Emerg Infect Dis. 22(8):1507–1509. doi: 10.3201/eid2208.160146. 27331418 PMC4982152

[CIT0132] Shevach EM. 2009. Mechanisms of foxp3+ T regulatory cell-mediated suppression. Immunity. 30(5):636–645. doi: 10.1016/j.immuni.2009.04.010. 19464986

[CIT0133] Shrestha A, Sadeyen J-R, Lukosaityte D, Chang P, Smith A, Van Hulten M, Iqbal M. 2021. Selectively targeting haemagglutinin antigen to chicken CD83 receptor induces faster and stronger immunity against avian influenza. NPJ Vaccines. 6(1):90., doi: 10.1038/s41541-021-00350-3. 34267228 PMC8282863

[CIT0134] Shrestha A, Sadeyen JR, Lukosaityte D, Chang P, Van Hulten M, Iqbal M. 2021. Targeting Haemagglutinin Antigen of Avian Influenza Virus to Chicken Immune Cell Receptors Dec205 and CD11c Induces Differential Immune-Potentiating Responses. Vaccines (Basel. 9(7):784. doi: 10.3390/vaccines9070784. 34358200 PMC8310205

[CIT0135] Sica A, Mantovani A. 2012. Macrophage plasticity and polarization: in vivo veritas. J Clin Invest. 122(3):787–795. doi: 10.1172/JCI59643. 22378047 PMC3287223

[CIT0136] Sridhar S, Begom S, Bermingham A, Hoschler K, Adamson W, Carman W, Bean T, Barclay W, Deeks JJ, Lalvani A, et al. 2013. Cellular immune correlates of protection against symptomatic pandemic influenza. Nat Med. 19(10):1305–1312. doi: 10.1038/nm.3350. 24056771

[CIT0137] Stein-Streilein J, Guffee J. 1986. In vivo treatment of mice and hamsters with antibodies to asialo GM1 increases morbidity and mortality to pulmonary influenza infection. J Immunol. 136(4):1435–1441. 3944461

[CIT0138] Stoop JN, van der Molen RG, Baan CC, van der Laan LJW, Kuipers EJ, Kusters JG, Janssen HLA. 2005. Regulatory T cells contribute to the impaired immune response in patients with chronic hepatitis B virus infection. Hepatology. 41(4):771–778., doi: 10.1002/hep.20649. 15791617

[CIT0139] Straub C, Neulen M-L, Sperling B, Windau K, Zechmann M, Jansen CA, Viertlboeck BC, Göbel TW. 2013. Chicken NK cell receptors. Dev Comp Immunol. 41(3):324–333., doi: 10.1016/j.dci.2013.03.013. 23542703

[CIT0140] Sun X, Xu X, Liu Q, Liang D, Li C, He Q, Jiang J, Cui Y, Li J, Zheng L, et al. 2013. Evidence of avian-like H9N2 influenza A virus among dogs in Guangxi, China. Infect Genet Evol. 20:471–475. doi: 10.1016/j.meegid.2013.10.012. 24161411

[CIT0141] Survashe B, Altken I. 1977. Further observations on functional deletion of paraocular glands in the fowl (Galius domesticus). Res Vet Sci. 23(2):217–223. doi: 10.1016/S0034-5288(18)33157-6. 563096

[CIT0142] Suvas S, Kumaraguru U, Pack CD, Lee S, Rouse BT. 2003. CD4 + CD25+ T cells regulate virus-specific primary and memory CD8+ T cell responses. J Exp Med. 198(6):889–901. doi: 10.1084/jem.20030171. 12975455 PMC2194203

[CIT0143] Suzuki T, Kawaguchi A, Ainai A, et al. 2015. Relationship of the quaternary structure of human secretory IgA to neutralization of influenza virus. Proceedings of the National Academy of Sciences of the United States of America 112:p. 7809–7814. doi: 10.1073/pnas.1503885112. PMC4485102 26056267

[CIT0144] Swayne DE, Kapczynski D. 2008. Strategies and challenges for eliciting immunity against avian influenza virus in birds. Immunol Rev. 225:314–331. doi: 10.1111/j.1600-065X.2008.00668.x. 18837791

[CIT0145] Szabo SJ, Sullivan BM, Peng SL, Glimcher LH. 2003. Molecular mechanisms regulating Th1 immune responses. Annu Rev Immunol. 21:713–758. doi: 10.1146/annurev.immunol.21.120601.140942. 12500979

[CIT0146] Szenberg A, Warner NL. 1962. Dissociation of immunological responsiveness in fowls with hormonally arrested development of lymphoid tissues. Nature. 194(4824):146–147. doi: 10.1038/194146b0.13874945

[CIT0147] Takeuchi O, Akira S. 2009. Innate immunity to virus infection. Immunol Rev. 227(1):75–86. doi: 10.1111/j.1600-065X.2008.00737.x. 19120477 PMC5489343

[CIT0148] Tamura S, Funato H, Hirabayashi Y, Suzuki Y, Nagamine T, Aizawa C, Kurata T. 1991. Cross-protection against influenza A virus infection by passively transferred respiratory tract IgA antibodies to different hemagglutinin molecules. Eur J Immunol. 21(6):1337–1344., doi: 10.1002/eji.1830210602. 1646112

[CIT0149] Tanaka A, Sakaguchi S. 2017. Regulatory T cells in cancer immunotherapy. Cell Res. 27(1):109–118. doi: 10.1038/cr.2016.151. 27995907 PMC5223231

[CIT0150] Taylor PR, Martinez-Pomares L, Stacey M, Lin HH, Brown GD, Gordon S. 2005. Macrophage receptors and immune recognition. Annu Rev Immunol. 23:901–944. doi: 10.1146/annurev.immunol.23.021704.115816. 15771589

[CIT0151] Teng QY, Zhou JY, Wu JJ, Guo JQ, Shen HG. 2006. Characterization of chicken interleukin 2 receptor alpha chain, a homolog to mammalian CD25. FEBS Lett. 580(17):4274–4281. doi: 10.1016/j.febslet.2006.06.044. 16831435

[CIT0152] Toro H, Fernandez I. 1994. Avian infectious bronchitis: specific lachrymal IgA level and resistance against challenge. Zentralbl Veterinarmed B. 41(7-8):467–472. doi: 10.1111/j.1439-0450.1994.tb00252.x. 7701859

[CIT0153] Uyeki TM, Nguyen DC, Rowe T, Lu X, Hu-Primmer J, Huynh LP, Hang NLK, Katz JM. 2012. Seroprevalence of antibodies to avian influenza A (H5) and A (H9) viruses among market poultry workers, Hanoi, Vietnam, 2001. PLoS One. 7(8):e43948., doi: 10.1371/journal.pone.0043948. 22928049 PMC3424239

[CIT0154] Vainio O, Koch C, Toivanen A. 1984. B-L antigens (class II) of the chicken major histocompatibility complex control T-B cell interaction. Immunogenetics. 19(2):131–140. doi: 10.1007/BF00387856. 6607882

[CIT0155] Van Campen H, Easterday BC, Hinshaw VS. 1989. Virulent avian influenza A viruses: their effect on avian lymphocytes and macrophages in vivo and in vitro. J Gen Virol. 70(11):2887–2895. doi: 10.1099/0022-1317-70-11-2887. 2685173

[CIT0156] Vervelde L, Matthijs MG, van Haarlem DA, de Wit JJ, Jansen CA. 2013. Rapid NK-cell activation in chicken after infection with infectious bronchitis virus M41. Vet Immunol Immunopathol. 151(3-4):337–341. doi: 10.1016/j.vetimm.2012.11.012. 23245429 PMC7112528

[CIT0157] Voskoboinik I, Smyth MJ, Trapani JA. 2006. Perforin-mediated target-cell death and immune homeostasis. Nat Rev Immunol. 6(12):940–952. doi: 10.1038/nri1983. 17124515

[CIT0158] Wang J, Tang C, Wang Q, Li R, Chen Z, Han X, Wang J, Xu X. 2015. Apoptosis induction and release of inflammatory cytokines in the oviduct of egg-laying hens experimentally infected with H9N2 avian influenza virus. Vet Microbiol. 177(3-4):302–314., doi: 10.1016/j.vetmic.2015.04.005. 25911114

[CIT0159] Warner NL, Szenberg A, Burnet FM. 1962. The immunological role of different lymphoid organs in the chicken. I. Dissociation of immunological responsiveness. Aust J Exp Biol Med Sci. 40:373–387. doi: 10.1038/icb.1962.42. 13998953

[CIT0160] Warner NL, Szenberg A. 1962. Effect of neonatal thymectomy on the immune response in the chicken. Nature. 196:784–785. doi: 10.1038/196784a0. 13998954

[CIT0161] Webster RG, Bean WJ, Gorman OT, Chambers TM, Kawaoka Y. 1992. Evolution and ecology of influenza A viruses. Microbiol Rev. 56(1):152–179. doi: 10.1128/mr.56.1.152-179.1992. 1579108 PMC372859

[CIT0162] Workman CJ, Szymczak-Workman AL, Collison LW, Pillai MR, Vignali DA. 2009. The development and function of regulatory T cells. Cell Mol Life Sci. 66(16):2603–2622. doi: 10.1007/s00018-009-0026-2. 19390784 PMC2715449

[CIT0163] Wu R, Chen Q, Zheng L, Chen J, Sui Z, Guan Y, Chen Z. 2009. Generation and evaluation of an H9N1 influenza vaccine derived by reverse genetics that allows utilization of a DIVA strategy for control of H9N2 avian influenza. Arch Virol. 154(8):1203–1210., doi: 10.1007/s00705-009-0425-6. 19543688

[CIT0164] Wu Y, Wu Y, Tefsen B, Shi Y, Gao GF. 2014. Bat-derived influenza-like viruses H17N10 and H18N11. Trends Microbiol. 22(4):183–191. doi: 10.1016/j.tim.2014.01.010. 24582528 PMC7127364

[CIT0165] Wu Z, Hu T, Kaiser P. 2011. Chicken CCR6 and CCR7 are markers for immature and mature dendritic cells respectively. Dev Comp Immunol. 35(5):563–567. doi: 10.1016/j.dci.2010.12.015. 21195108

[CIT0166] Wu Z, Rothwell L, Young JR, Kaufman J, Butter C, Kaiser P. 2010. Generation and characterization of chicken bone marrow-derived dendritic cells. Immunology. 129(1):133–145. doi: 10.1111/j.1365-2567.2009.03129.x. 19909375 PMC2807494

[CIT0167] Xing Z, Cardona CJ, Adams S, Yang Z, Li J, Perez D, Woolcock PR. 2009. Differential regulation of antiviral and proinflammatory cytokines and suppression of Fas-mediated apoptosis by NS1 of H9N2 avian influenza virus in chicken macrophages. J Gen Virol. 90(Pt 5):1109–1118., doi: 10.1099/vir.0.007518-0. 19264628

[CIT0168] Xing Z, Cardona CJ, Li J, Dao N, Tran T, Andrada J. 2008. Modulation of the immune responses in chickens by low-pathogenicity avian influenza virus H9N2. J Gen Virol. 89(Pt 5):1288–1299. doi: 10.1099/vir.0.83362-0. 18420808

[CIT0169] Xu L, Huang Y, Yang J, Van Der Meide PH, Levi M, Wahren B, Link H, Xiao B. 1999. Dendritic cell-derived nitric oxide is involved in IL-4-induced suppression of experimental allergic encephalomyelitis (EAE) in Lewis rats. Clin Exp Immunol. 118(1):115–121., doi: 10.1046/j.1365-2249.1999.01029.x. 10540168 PMC1905389

[CIT0170] Xu X, Qian J, Qin L, Li J, Xue C, Ding J, Wang W, Ding W, Yin R, Jin N, et al. 2020. Chimeric Newcastle Disease Virus-like Particles Containing DC-Binding Peptide-Fused Haemagglutinin Protect Chickens from Virulent Newcastle Disease Virus and H9N2 Avian Influenza Virus Challenge. Virol Sin. 35(4):455–467., doi: 10.1007/s12250-020-00199-1. 32274680 PMC7462956

[CIT0171] Yang J, Huang X, Liu Y, Zhao D, Han K, Zhang L, Li Y, Liu Q. 2020. Analysis of the microRNA expression profiles of chicken dendritic cells in response to H9N2 avian influenza virus infection. Vet Res. 51(1):132., doi: 10.1186/s13567-020-00856-z. 33069243 PMC7568386

[CIT0172] Yasuda M, Kajiwara E, Ekino S, Taura Y, Hirota Y, Horiuchi H, Matsuda H, Furusawa S. 2003. Immunobiology of chicken germinal center: i. Changes in surface Ig class expression in the chicken splenic germinal center after antigenic stimulation. Dev Comp Immunol. 27(2):159–166., doi: 10.1016/s0145-305x(02)00066-6. 12543129

[CIT0173] Yasuda M, Taura Y, Yokomizo Y, Ekino S. 1998. A comparative study of germinal center: fowls and mammals. Comp Immunol Microbiol Infect Dis. 21(3):179–189. doi: 10.1016/s0147-9571(98)00007-1. 9681241

[CIT0174] Zhao X, Dai J, Xiao X, Wu L, Zeng J, Sheng J, Su J, Chen X, Wang G, Li K, et al. 2014. PI3K/Akt signaling pathway modulates influenza virus induced mouse alveolar macrophage polarization to M1/M2b. PLoS One. 9(8):e104506. doi: 10.1371/journal.pone.0104506. 25105760 PMC4126709

[CIT0175] Zhirnov OP, Klenk HD. 2007. Control of apoptosis in influenza virus-infected cells by up-regulation of Akt and p53 signaling. Apoptosis. 12(8):1419–1432. doi: 10.1007/s10495-007-0071-y. 17468837

[CIT0176] Zhou G, Juang SW, Kane KP. 2013. NK cells exacerbate the pathology of influenza virus infection in mice. Eur J Immunol. 43(4):929–938. doi: 10.1002/eji.201242620. 23436540

[CIT0177] Zou J, Chang M, Nie P, Secombes CJ. 2009. Origin and evolution of the RIG-I like RNA helicase gene family. BMC Evol Biol. 9:85. doi: 10.1186/1471-2148-9-85. 19400936 PMC2686710

